# A scoping review of gender-based violence interventions conducted in Afghanistan

**DOI:** 10.1186/s12939-026-02830-1

**Published:** 2026-03-25

**Authors:** Wahiba Abu-Ras, Basil H. Aboul-Enein, Patricia J. Kelly, Dilshod Achilov

**Affiliations:** 1 School of Social Work, Adelphi University, Garden City, NY USA; 2 College of Arts & Sciences Health & Society Program, University of Massachusetts Dartmouth, 285 Old Westport Rd, North Dartmouth, MA 02747 USA; 3 London School of Hygiene & Tropical Medicine, Faculty of Public Health and Policy, 15-17 Tavistock Place, London, WC1H 9SH UK; 4 Thomas Jefferson University College of Nursing, 901 Walnut Street, Philadelphia, PA USA; 5 Department of Political Science, University of Massachusetts Dartmouth College of Arts & Sciences, 285 Old Westport Rd, North Dartmouth, MA 02747 USA

**Keywords:** Afghanistan, Violence against women, Intervention, Prevention, Gender-based violence

## Abstract

**Background:**

Gender-based violence (GBV) remains one of the most pervasive human rights and public health challenges worldwide, with disproportionate impacts on women and girls in conflict-affected and fragile states. Afghanistan represents one of the most restrictive environments for women’s rights, where structural, cultural, and political barriers severely limit the implementation and evaluation of GBV-prevention programs. This scoping review aims to identify and critically appraise GBV prevention and response interventions implemented in Afghanistan and assess their contribution to advancing gender and health equity.

**Methods:**

Following PRISMA-ScR guidelines and the PICOS framework, nine databases were systematically searched for studies published between 2010 and November 2025. Studies were included if they evaluated or described an intervention addressing GBV, intimate partner violence, or violence against Afghan women and girls. Data were charted by study design, target population, intervention type, theoretical foundation, and outcomes.

**Results:**

Seven studies met inclusion criteria, highlighting the limited evidence base in Afghanistan. Interventions included community development programs, school-based peace education, family strengthening initiatives, psychosocial counselling, and women’s economic empowerment. Reported outcomes showed improvements in gender-equitable attitudes, family relationships, and psychosocial wellbeing; however, cultural constraints and insecurity limited long-term impact.

**Conclusions:**

Despite sparse evidence, existing interventions suggest that context-sensitive, community-driven, and multi-sectoral approaches can promote gender equity and resilience. Expanding locally led GBV-prevention programs integrated into health and social systems can assist in reducing violence and achieving equitable health outcomes for Afghan women and families.

## Introduction

 Violence against women and girls (VAWG) continues to be a pervasive human rights issue globally, with its impacts intensified in settings marked by conflict, displacement, and entrenched patriarchal norms. In the context of humanitarian crises, VAWG exhibits both an increased frequency and heightened complexity. This phenomenon can be understood as a direct consequence of the disintegration of protective institutional frameworks and the subsequent deterioration of social order [1, 2]. Afghanistan acutely exemplifies these dynamics. Decades of war, political instability, and structural inequities have produced forms of everyday and institutional violence that compound women’s vulnerability [3]. The country’s protracted conflict since 1973 has contributed not only to direct forms of harm but also to widespread structural and symbolic violence limiting women’s agency, bodily autonomy, and access to essential services. Research on gender-based violence (GBV) in conflict zones highlights that when violence is institutionalized, it reinforces social hierarchies that limit women’s participation in health, education, and governance [4, 5]. The Afghanistan Independent Human Rights Commission documented an approximate 25% increase in violence against women, with Afghanistan ranked as the most dangerous country for women worldwide [6].

In this review, GBV refers broadly to violence directed at individuals based on gender, including physical, sexual, psychological, and economic harm. More specifically, GBV is defined as any act of gender-based violence that results in, or is likely to result in, physical, sexual, or mental harm or suffering to women. VAWG refers to any act of gender-based violence that results in, or is likely to result in, physical, sexual, or psychological harm or suffering to women, including threats of such acts, coercion, or arbitrary deprivation of liberty, whether occurring in public or in private life. Specifically, violence targeting women and girls as a gendered population [7–9]. Although GBV was used as a search umbrella term to capture variation in database indexing, the substantive focus of this review is on interventions addressing violence against women and girls in Afghanistan.

Since the Taliban’s takeover of Afghanistan in August 2021, their policies and practices have specifically targeted the choices, rights, and bodies of Afghan women and girls [10–12]. Multiple United Nations and humanitarian assessments confirm that these policies and practices have led to the near-total collapse of gender-sensitive health and protection services [13–17]. For example, the Afghan government systematically excluded women from public and political life, which not only negatively impacted Afghanistan’s progress in realizing the globally agreed Sustainable Development Goals (SDGs) but also reversed the fragile gains made on gender equality and women’s empowerment between 2001 and 2021 [18]. Instead, these extremist restrictions severely constrained women’s ability to influence decisions affecting their lives, undermining gender equality and exacerbating poverty and instability across the country. A recent World Health Organization (WHO) situation report confirms that the rollback of female participation in healthcare, particularly midwifery and psychosocial services, has left millions of women without access to maternal or trauma-related care, increasing inequities in both service delivery and health outcomes [19].

In just three years alone (2021–2024), the Taliban authorities imposed sweeping bans that excluded Afghan women from sports, higher education, most forms of employment, public spaces, independent travel, and participation in civic and professional institutions [13, 14, 20]. Although the Taliban justified these policies as religiously grounded, extensive scholarship in Islamic jurisprudence and the practices of many Muslim-majority countries, including highly conservative states (e.g., Iran, Saudi Arabia), demonstrate that such prohibitions are not mandated by Islamic teaching and do not reflect the broader diversity of Islamic legal and cultural interpretations [21]. The instrumentalization of religion in this context serves a political rather than theological purpose, aimed at controlling women’s mobility and silencing dissent [22, 23]. This mirrors broader patterns of “gendered authoritarianism” observed in conflict zones where women’s rights become a battleground for political legitimacy. Given this context, VAWG in Afghanistan is both widespread and normalized. Analyses of nationally representative data from the 2015 Afghanistan Demographic and Health Survey indicate that more than half of ever-married Afghan women have experienced at least one form of intimate partner violence [24].

Women routinely face emotional, physical, and sexual violence in contexts where disclosure carries risks of stigmatization, retaliation, or social exclusion [25]. Social norms that position women as subordinate to men remain pervasive across Afghan communities [26]. Broader structural constraints, including limited access to education, constrained mobility, gendered restrictions on public engagement, and the near absence of institutional protection systems, often reinforce these norms. Domestic violence is perceived as a family affair, leading to widespread underreporting. The Elimination of Violence Against Women Law (EVAW), passed in 2009, criminalized 22 acts of violence but was formally dismantled in 2021. This was a major step backward, effectively removing one of the few institutional mechanisms for redress despite more than 4,000 prosecutions [6]. In humanitarian and conflict-affected settings, such legal regression contributes to what Stark et al. [2] describe as “policy-induced vulnerability,” wherein women’s exposure to violence is exacerbated by state collapse and patriarchal governance structures.

Evidence from Afghanistan highlights not only the prevalence of IPV but also its severe consequences. Analyses of the 2015 Afghanistan Demographic and Health Survey reveal that physical IPV is significantly associated with adverse neonatal outcomes, including an increased hazard of neonatal mortality, particularly among women exposed to severe forms of violence [24]. Additional studies have reported associations between IPV and childhood morbidity, under-five mortality, maternal mental health disorders, and compromised child development [25, 27]. While physical violence has received extensive research attention, emotional violence, involving manipulation, degradation, or control, remains largely underexamined. Psychological scores are more strongly associated with adverse health outcomes than physical scores [28]. Similar findings have emerged from other conflict settings such as Syria, Myanmar, and the Democratic Republic of Congo, where GBV has been shown to increase women’s risk of depression, obstetric complications, and suicide, underscoring its direct role in deepening health inequities [2, 5, 29].

The political transition of 2021 has further eroded the mental health infrastructure. Studies in this area have documented widespread trauma among Afghan women navigating both domestic violence and broader conflict-related stressors, compounded by the absence of safe disclosure spaces or institutional pathways for support [25, 30]. Afghanistan’s maternal mortality ratio, among the world’s highest, intersects with severe shortages of female health providers. While most women accessed health care for abuse-related concerns, less than half reported abuse to providers or were asked about injury context [6]. This gap reflects inadequate physician education on GBV, the absence of assessment tools, unreliable reporting systems, and cultural taboos. These barriers reflect a broader humanitarian health gap, in which GBV remains largely unincorporated into primary care and emergency response frameworks. In conflict zones, the absence of routine GBV screening and survivor-centered care perpetuates the invisibility of violence as a health issue, reinforcing structural inequities across gender and class lines [1]. Evidence from humanitarian and conflict-affected settings demonstrates that access to GBV services is socially stratified, with rural, economically marginalized, and socially isolated women facing greater barriers to disclosure and support [2, 31](Stark et al., 2022; WHO, 2013). In Afghanistan specifically, women’s restricted mobility, stigma, and dependence on male-controlled resources further limit help-seeking pathways [25]. Without systematic assessment and referral mechanisms within primary healthcare settings, women with fewer financial or social resources are less likely to access protection services, reinforcing structural inequities across socioeconomic strata. In this context, preventive interventions targeting VAWG encounter profound structural, cultural, and logistical barriers.

Despite urgency, intervention-focused research in Afghanistan remains limited, fragmented, and severely constrained by security challenges, ethical concerns, and restricted community access. Studies comparing community-level VAWG prevention mechanisms show Afghanistan exhibits some of the weakest activation of community-led prevention structures, particularly due to norms framing violence as private and discouraging collective accountability [26]. Much existing evidence documents prevalence rather than systematically evaluating prevention, psychosocial support, or health system responses. Emerging evidence suggests maternal and reproductive health platforms, particularly antenatal care and skilled birth attendance, may serve as feasible IPV screening, support, and referral entry points, given their moderating effects on physical violence health consequences [24]. Similarly, studies from other conflict-affected regions demonstrate that integrated economic and psychosocial interventions, including combining cash transfers with social norm change programming, can significantly reduce IPV and enhance women’s well-being when adapted to local contexts [32]. These findings suggest a critical opportunity to adapt similar approaches for Afghanistan, emphasizing culturally sensitive, community-led delivery models.

To this end, studying Afghanistan is therefore essential not only for national policy and humanitarian programming, but also for the broader global evidence base on GBV prevention in crisis-affected settings. Afghanistan represents an extreme yet critically understudied case in which legal precarity, state fragility, exposure to conflict, and gendered restrictions converge. From a global equity perspective, Afghanistan exemplifies what Kruk et al. [33] describe as “extreme health inequity,” in which social determinants such as gender, conflict, and poverty converge to produce profound disparities in survival and agency. Understanding how interventions operate and under what conditions they succeed or fail is pivotal to developing equitable and sustainable prevention models in resource-constrained and politically restrictive contexts.

To address these gaps, this scoping review synthesizes existing research on preventive interventions addressing violence against women and girls in Afghanistan. The review aims to generate a comprehensive understanding of intervention models, mechanisms, and outcomes within this unique sociopolitical landscape, while contributing to global commitments toward gender equality and the violence-prevention objectives articulated in the Sustainable Development Goals (particularly SDG 5). By situating Afghanistan within the broader humanitarian literature, this review also seeks to illuminate how VAWG functions as a determinant of health inequity, emphasizing the urgent need for context-adapted, evidence-based, and equity-oriented approaches to GBV prevention in fragile settings.

### Historical context of Afghan women’s struggles

Afghanistan’s modern history has been marked by repeated cycles of reform, foreign intervention, and sociopolitical regression, with women’s rights serving as a persistent battleground. During the constitutional monarchy under King Zahir Shah (1933–1973), women made modest yet meaningful gains in education and public participation, particularly in urban centers such as Kabul and Herat. The 1964 Constitution expanded women’s suffrage and parliamentary representation [34, 35], reflecting an early effort toward gender inclusion in governance.

The 1973 coup and subsequent Soviet invasion in 1979 fundamentally altered Afghanistan’s political and social landscape. The People’s Democratic Party of Afghanistan introduced modernization policies, including compulsory education for girls and restrictions on child marriage, but these reforms triggered backlash from conservative factions, resulting in gender-targeted violence against women active in public life [36, 37]. Prolonged conflict displaced millions and weakened health and education infrastructure; by the late 1980s, nearly six million Afghans had fled to neighboring countries [38].

The Taliban’s first rule (1996–2001) imposed sweeping restrictions on women’s mobility, education, and employment, institutionalizing surveillance through the Ministry for the Promotion of Virtue and Prevention of Vice [10, 15, 39]. Girls’ schools were closed, women required male guardians for movement, and maternal mortality rates rose to among the highest globally, exceeding 1,600 deaths per 100,000 live births [7, 40]. After 2001, constitutional reforms and international support reopened opportunities for women’s participation. The 2004 Constitution mandated female parliamentary representation, and the Elimination of Violence Against Women law in 2009 criminalized multiple forms of abuse and established protection mechanisms. However, progress remained uneven, urban-centered, and donor-dependent, with rural and displaced women continuing to face barriers to justice and services [41].

By the late 2010s, community-based GBV prevention and psychosocial programs had expanded, although tensions persisted between women’s rights advocates and conservative authorities [41, 42]. These tensions underscored the fragility of reforms that relied heavily on political support and external funding rather than durable institutional transformation.

The Taliban’s return to power in 2021 reversed many of these gains. More than sixty decrees between 2021 and 2024 restricted women’s education, employment, and mobility [13, 14]. The dissolution of the Ministry of Women’s Affairs and the reinstatement of the Ministry for the Promotion of Virtue and Prevention of Vice marked a shift from rights-based governance to coercive control. Afghanistan remains the only country where girls are barred from secondary and tertiary education, a situation described as “gender apartheid” [38].

The humanitarian consequences have been severe. Female participation in the healthcare workforce has declined substantially, limiting maternal and psychosocial care availability [40, 43]. Reports indicate increased gender-based violence amid economic collapse, displacement, and weakened accountability systems [15, 16]. The closure of shelters and erosion of legal protections have further restricted survivors’ access to justice.

This historical trajectory demonstrates how gender equality in Afghanistan has been repeatedly advanced and reversed through shifts in governance and conflict. Such volatility underscores that GBV prevention efforts must be understood within broader patterns of institutional fragility, structural violence, and constrained service infrastructure in conflict-affected settings.

## Methods

### Search procedures

In spite of the interchangeable use of GBV and VAWG, both terms imply that VAWG often occurs from gender inequality and discrimination against women in patriarchal societies. Therefore, inequality between the sexes is structural because constructions of femininity and masculinity are symbolic and reinforced by cultural norms [44]. Therefore, of note is that while the term used in this review is GBV, other terms used in the literature include violence against women (VAW) and violence against women and girls (VAWG), which were included as search terms in this review. To identify intervention strategies conducted in Afghanistan to prevent GBV/VAWG/VAW, Population, Intervention, Comparison, Outcomes, and Study (PICOS) formatting was utilized to develop inclusion and exclusion criteria for this review (Table 1). The PICOS design guidelines were incorporated to develop the research question: ‘Do female and/or male populations of all age groups” (P) that are offered interventions focusing on prevention of GBV/VAWG/VAW (I) have improved outcomes (O) compared with those that do not participate in interventions focusing on prevention of GBV/VAWG/VAW (C)?’ and subsequent inclusion and exclusion criteria (see Table 1). The search was conducted in late 2025, and databases were searched from 2010 through November 2025.

**Table 1:** Inclusion and exclusion criteria

Date Range	Inclusion	Exclusion
	Between 2010 and November 2025	-
Research Design	Intervention studies, RCTs, quasi-experimental, qualitative studies with intervention component assessed	Non-interventional studies. Systematic, scoping, rapid and literature reviews. Commentaries, narratives, protocols, editorial communications, opinion pieces, conference papers, white papers, grey literature, theses, dissertations, government reports, guidance documents.
Sources	Peer reviewed empirical evidence	-
Languages	English	-
Geographic location	Afghanistan	-
Groups	1. female and/or male populations 2. Adults > 18 years 3. children and young people aged 0-17years	Afghani Diaspora and communities residing outside the geographic location of interest
Focus of study	1. Interventions focusing on prevention of VAW 2. Interventions focusing on education about VAW to assist in its prevention 3. Digital and face-to-face interventions	1. Clinical interventions not focusing on VAWG 2. Clinical or psychological treatment and care of females after experiencing violence

### Selection criteria

**Fig. 1:**
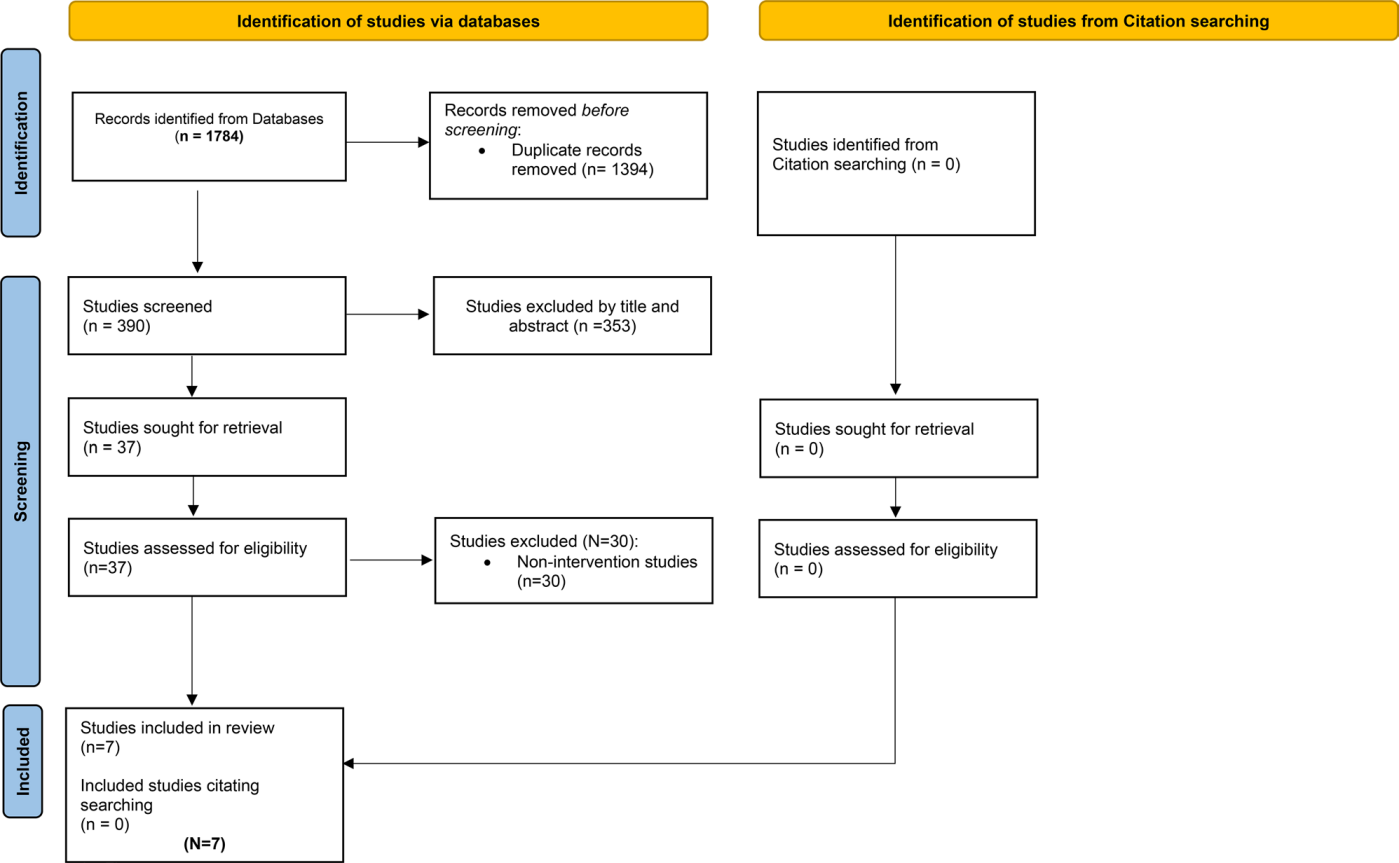
PRISMA flow diagram

For this scoping review, the Preferred Reporting Items for Systematic Reviews and Meta-Analyses for Scoping Reviews (PRISMA-ScR) guidelines were utilized [45]. Databases searched were Scopus, MEDLINE, CINAHL, Embase via OVID, Web of Science, PsycINFO, PubMed, ASSIA, and Google Scholar (Table 2). The indexing systems of each respective database adapted the search strategy. Search terms combined techniques, such as Medical Subject Headings (MeSH) keywords, phrases, and Boolean operators (Table 2). PJK and BA-E screened titles and abstracts for relevance. BA-E worked with Rayyan QCRI software to assist in the screening process [46]. Consultation with an independent reviewer resolved any potential disagreements to reach a consensus. PJK and BA-E made the final decisions about inclusion and documented reasons for exclusion. Figure 1 provides the PRISMA flowchart leading to selected studies for this review. Because methodological quality assessment is not a prerequisite for scoping reviews, an appraisal of the included studies was not included [47].

**Table 2:** Electronic databases used with relevant search period and terms

Databases	Search Period	MeSH keywords, terms, phrases, and Boolean operators
PubMed, MEDLINE, CINAHL, Embase via OVID, Web of Science, PsycInfo, SCOPUS, ASSIA, Google Scholar	2010 and November 2025	(violence against women), OR (VAW), OR (gender based violence), OR (GBV), OR (domestic violence [MeSH Terms]) OR (domestic violence) OR (intimate partner violence [MeSH Terms]) OR (intimate partner violence) OR (spousal violence[MeSH Terms]) OR (spouse violence) OR (family violence), OR (physical abuse [MeSH Terms]) OR (physical abuse) OR (physical violence [MeSH Terms]) OR (physical violence) OR (battered women) OR (battered females) OR (emotional violence[MeSH Terms]) OR (emotions violence) OR (emotions abuse[MeSH Terms]) OR (emotional abuse) OR (psychological violence [MeSH Terms]) OR (conjugal violence) OR (dating violence) OR (psychological violence) OR (psychological abuse [MeSH Terms]) OR (psychological abuse) OR (sexual violence [MeSH Terms]) OR (sex violence) OR (sex abuse[MeSH Terms]) OR (sex abuse) OR (harassment [MeSH Terms]) OR (harassment) OR (intimidation[MeSH Terms]) OR (intimidation) OR (sexual assault [MeSH Terms]) OR (sexual assault) OR (sexual coercion[MeSH Terms]) OR (sexual coercion) OR (rape [MeSH Terms]) OR rape) OR (cyber-stalking) OR (cyber violence) OR (female genital mutilation) OR (child marriage) AND ((disclosure [MeSH Terms]) OR (disclosure) OR (help seeking [MeSH Terms]) OR (help seeking) OR (service utilization [MeSH Terms]) OR (service utilization) OR (prevention), OR (interventions) AND Afghanistan OR Afghani* [MeSH Terms]

The review explored the characteristics of interventions, target audiences, and program outcomes and tabulated the included studies (see Table 3).

**Table 3:** Characteristics of included studies -Afghanistan VAW (*N* = 7)

Author, Year; Country	Research Question	Population/ Sample	Study Design	Content/ Training	Results	Discussion
Beath, et al., 2013	Can a community driven development initiative *(National Solidarity Program -NSP*) promote women’s empowerment?	50 villages from 10 districts, half received development program	Cross-over RCT	Bundled development project with both elected gender- balanced local councils, allocation of funds; rehabilitation of infrastructure, human capital development (training, literacy); at least 1 project targeting women	In NSP villages, men & women more likely to report increase in presence of well-respected women in community; women no more likely to report increased frequency of socialization outside of household, increase in number of times outside of compound; NSP had virtually no effect on women’s position in family, no change in agency over money/assets; no effect on attitudes toward women in public sphere; increase in income generating activities	Women’s involvement in community-level decision-making mandated by NSP, making men more receptive to women’s involvement in community life; no carry-over of positive effects to areas not directly linked to program’s interventions (role of women in family or broader public sphere)
Corboz, et al., 2019	Can a community-based intervention *Help the Afghan Children (HTAC)* change harmful social norms & practices related to gender & use of violence against children in conflict resolution?	10 boys & 10 girls) school, with students from grade 7–10; total of 361 boys, 373 girls	One-group pre-post evaluation	2-year school-based peace education program with teachers, parents, community members using Help the Afghan Children’s theory of change delivered in 95 30-minute session by 50 trained teachers; radio messages	Significant reduction in boys’ and girls’ reports of experiencing physical punishment, experience of peer violence victimization or perpetrating violence, significantly more equitable gender attitudes, significantly less violence-supportive attitudes at endline than baseline, slower depression scores	Results suggest that conducting peace education with children in schools, alongside community components for adults with broader messaging about women’s & children’s rights, prevention of violence can lead to reduction violence
Ehsani, et al., 2024	What was MH & psychosocial support (MHPSS) effect of hotline?	924 calls x 6 months; 32 concluded cases, 3 in-depth case reports	Descriptive program evaluation	Free hotline with 2 male/2 female counselors providing immediate and short-term problem-solving, counseling skills, MHPSS services	Main themes: GBV social difficulties, family conflicts, substance use; hotline was cost-effective, well-received; MH significantly improved after average of 6 sessions; limitation was many women had to use husband’s phone to make calls	Hotline effective in providing service to women with access to mobile phone; results highlight importance of accessible MHPSS interventions; must address accessibility to ensure all women have access to services
Gibbs, et al., 2020	Can the *Women for Women International* economic & social empowerment program reduce women’s IPV experiences & depression?	1477 females, 18–49 years old, with minimal education, extreme poverty from 6 conflict-afflicted communities	RCT plus qualitative interviews	Rights-based program of 90–180 min/week x 1 year, delivered to groups of 25 women; content on numeracy, business skills. empowerment, hands-on vocational training, cash stipends, strategies to save money, referrals to health, legal, financial services,	Primary outcomes of physical IPV, severe IPV, depressive symptoms in hypothesized direction, but no significant impact; also reduced food insecurity, higher earnings, savings, less gender-inequitable attitudes, more household decision-making, increased mobility	Results indicate potential for progress on economic empowerment; qual interviews provide important pointers for future intervention strengthening
Haar, et al., 2020	Test the feasibility of implementation, initial effective of *Strong Families*, in improving child behavior, family functioning in families living in Afghanistan.	72 female caregivers & children 8–12 years old years	One-group pre-post intervention	3 session family skills program, total 5 h, parental challenges/dealing with stress; showing love with limits; communication skills; encouraging good/discouraging bad behaviors, future goals for children; family values	Strengths & Difficulties score reduced significantly, 17.8 at pre- test to 12.9 at post-test, 10.6 at second follow-up, no difference in gender, most noticeable in those with ighest scores at baseline; scores on parenting practices/ family functioning scores improved significantly post program	Program effective & feasible in resource-limited setting, positively improved child MH, parenting practices, family adjustment skills; results call for further validation of impact assessment and outcome evaluation
Manell, Ahmad & Ahmad, 2018	Examines potential for narrative storytelling to support women’s MH/ suffering caused by GBV	20 women living in safe houses for GBV, plus 8 safe house staff	Qual study	Symbolic interactionist perspective used to analyze semi-structured interviews	Storytelling under supportive conditions perceived as valuable experience that could help formulate positive social identities, challenge broader social structures; supportive conditions=presence of sympathetic, non-judgmental listener, supportive social environment	Findings offer alternative to bio-medical models of MH support for women experiencing GBV in high-prevalence settings
Tomlinson, et al. 2020	Evaluate feasibility of delivering a maternal mental health service in resource constrained setting	131 women PP women from 2 health facilities scoring > 12 on PHQ-9 screening instrument	Feasibility study	Psychological intervention, adaptation of *Thinking* *Healthy Program for PPD*, cognitive behavioral approach adapted for the Afghan context with stakeholder input	All 131 women who scored above the PHQ-9 cut-off of 12 signed consent; 72 (55%) participated in first session, number reduced weekly to final session with 47 (31%) in attendance; completers had average decrease of 13 points in depressions scores; all scored below study cut-off score of 12	In poorly resourced environments, with high PPD prevalence, shift from specialist to primary care-level intervention may be viable way to provide maternal MH care, integrate into existing child health programs

### Ethics declaration

Given the nature of this scoping review, no ethical oversight was found to be necessary for this review and, therefore, no institutional review board was acquired.

## Results

Seven intervention-focused studies met inclusion criteria, published between 2013 and 2024 [48–54]. Interventions spanned the prevention continuum, including primary prevention programs targeting social norms and family functioning, secondary prevention interventions addressing identified psychosocial risk, and tertiary response mechanisms providing direct support to survivors.

### Levels of prevention

#### Primary prevention interventions

Three studies focused on primary prevention through norm change, governance participation, and family strengthening [49, 51, 53].

The National Solidarity Program [51] implemented gender-balanced village development councils supported by block grants, requiring at least one project to benefit women. The intervention aimed to enhance women’s visibility and participation in community decision-making. Results indicated increased income-generating activities and greater recognition of respected women in communities. However, there were limited changes in women’s mobility or intra-household authority.

The school- and community-based peace education program [53] delivered 66 structured lessons emphasizing nonviolent conflict resolution, gender equity, and social tolerance. Community components included peace committees and radio programming. Significant reductions were observed in peer violence victimization and perpetration, as well as improved gender-equitable attitudes and reduced violence-supportive beliefs.

The Strong Families program [49] provided five contact hours of family skills training over three weeks. The intervention targeted stress management, communication, and positive parenting practices. Post-intervention assessments demonstrated significant improvements in parenting practices and family functioning, with greater gains among participants with higher baseline risk.

Collectively, primary prevention interventions primarily influenced normative attitudes, peer violence, and family-level functioning, while structural shifts in gender power dynamics were more limited.

#### Secondary prevention intervention

One study addressed secondary prevention through targeted mental health intervention [50]. The Thinking Healthy Program, adapted for Afghanistan [50], targeted women screening positive for postpartum depression in primary healthcare settings. The cognitive-behavioral intervention focused on structured problem-solving and behavioral activation. Among women who completed the intervention, significant reductions in depressive symptoms were observed. However, attrition exceeded 70%, limiting generalizability.

This intervention demonstrates that facility-based psychosocial approaches can yield measurable improvements in mental health, although sustained engagement remains challenging.

#### Tertiary prevention and response interventions

Two studies focused on tertiary prevention and direct survivor support [52, 54]. The mental health and psychosocial support hotline [54] provided daytime counseling services over a six-month period, receiving 924 calls. Counseling addressed gender-based violence, family conflict, and substance use. Participants demonstrated significant improvements in mental health following an average of six sessions. A key operational limitation was that many women relied on male-controlled phones, which constrained confidentiality and potentially limited access.

The storytelling intervention [52] was conducted in safe houses and facilitated narrative sharing of gender-based violence experiences. Participants reported the intervention as supportive and empowering, highlighting the importance of safe spaces for collective validation and psychosocial processing.

Tertiary interventions primarily relied on confidential support, narrative processing, and short-term psychosocial stabilization rather than on structural change.

### Outcome domains and measures

The included studies assessed multiple outcome domains related to violence exposure, psychosocial wellbeing, relational functioning, and economic empowerment. Forms of violence measured varied across interventions. Physical intimate partner violence and severe intimate partner violence were assessed in the randomized trial [48]. Peer violence victimization and perpetration, as well as violence-supportive attitudes and acceptance of physical punishment, were measured in the school-based peace education intervention [53].

Other studies assessed indirect or related indicators of gender-based violence risk. Beath et al. [51] examined women’s participation in income-generating activities, presence in community governance structures, and mobility as indicators of social empowerment. Haar et al. [49] measured parenting practices and family functioning as relational risk and protective factors. Mental health outcomes were assessed in both secondary and tertiary prevention interventions. Tomlinson et al. [50] evaluated depressive symptoms using standardized screening instruments among women screening positive for postpartum depression. While Ehsani et al. [54] reported improvements in psychological well-being following hotline counseling sessions. The storytelling intervention [52] focused on qualitative psychosocial outcomes, including perceived emotional relief and collective validation of lived experiences.

Across studies, outcome measurement approaches included randomized controlled trial designs, interrupted time series analysis, pre-post evaluations, program utilization statistics, and qualitative reporting. Few studies assessed long-term changes in violence exposure, and mediation analyses examining mechanisms of change were not formally conducted.

### Synthesis across prevention levels

Across prevention levels, interventions were delivered through schools, community councils, family sessions, primary healthcare facilities, safe houses, and telephonic platforms. Outcome measures included intimate partner violence exposure, peer violence, gender attitudes, depressive symptoms, family functioning, and economic indicators. While several interventions demonstrated improvements in psychosocial well-being and gender-equitable attitudes, reductions in intimate partner violence were limited or statistically nonsignificant in most cases [48]. Overall, primary prevention programs showed the most consistent shifts in normative and relational outcomes, secondary prevention demonstrated targeted mental health benefits with retention challenges, and tertiary interventions provided feasible psychosocial support under constrained conditions.

### Mechanisms of change

Across the included studies, intervention mechanisms were articulated through social norm transformation, economic strengthening, psychosocial skill development, participatory governance, and confidential support pathways. While not all studies formally tested mediators, authors described explicit theories of change underpinning intervention design.

Primary prevention interventions largely operate through social norms and behavior change mechanisms. The school-based peace education and community intervention described by Corboz et al. [53] was grounded in a whole-community theory of change, emphasizing that sustained exposure to non-violent conflict resolution training, positive role modeling, and community dialogue would reduce acceptance of violence and shift gender-equitable attitudes. The inclusion of peace committees and community radio discussions extended these mechanisms beyond classrooms, reinforcing normative messaging across household and community structures. Reported reductions in peer violence, physical punishment, and violence-supportive attitudes were consistent with these hypothesized norm-change pathways [53].

Similarly, the National Solidarity Program evaluated by Beath et al. [51] incorporated participatory governance mechanisms, including gender-balanced village development councils and mandatory inclusion of projects benefiting women. The program theorized that institutionalizing women’s visibility and participation in local decision-making structures would increase social legitimacy and potentially influence intra-community perceptions of women’s roles. Although improvements were observed in women’s participation in income-generating activities and in their presence in public forums, changes in intra-household power dynamics were limited, suggesting that participatory governance mechanisms may influence public visibility more readily than private household authority Beath et al. [51].

Economic and social empowerment programming, most notably the intervention evaluated by Gibbs et al. [48], combined vocational skills training, financial literacy education, peer support groups, and cash stipends. The intervention was designed to operate through increased economic capacity, enhanced decision-making autonomy, and collective empowerment processes. While statistically significant reductions in intimate partner violence were not observed, improvements in food security, savings, mobility, and gender-equitable attitudes were reported, indicating partial activation of the theorized economic empowerment pathways [48].

Secondary prevention efforts targeting mental health operate through cognitive-behavioral mechanisms. The Thinking Healthy Program, adapted for Afghanistan by Tomlinson et al. [50], utilized structured cognitive restructuring, behavioral activation, and problem-solving techniques to address depressive symptoms among women screening positive for postpartum depression. Significant symptom reduction among intervention completers suggests that cognitive-behavioral skill acquisition functioned as an effective mechanism for psychosocial improvement, although high attrition limited broader inference [50].

Tertiary interventions emphasized safe disclosure and psychosocial support pathways. The mental health and psychosocial support hotline evaluated by Ehsani et al. [54] provided immediate counseling, short-term problem-solving strategies, and referral guidance. Its mechanism is centered on accessibility, confidentiality, and rapid psychosocial response. Reported improvements in mental health outcomes suggest that structured telephonic counseling can function as a viable support mechanism in contexts of restricted mobility, though reliance on male-controlled phones constrained privacy for some participants [54]. Likewise, the storytelling intervention described by [52] functioned through narrative processing and collective validation of lived experiences within safe house environments, enabling participants to reframe traumatic experiences in supportive group contexts.

Overall, while mechanisms were described within individual intervention models, few studies empirically tested mediation pathways or examined long-term structural change. The evidence, therefore, reflects program-level theories of change rather than experimentally validated mechanism analyses.

## Discussion

This scoping review synthesizes a small and heterogeneous body of evidence on gender-based violence (GBV) prevention and response interventions implemented in Afghanistan. Given that only seven studies met the inclusion criteria, and that study designs, outcomes, and populations varied considerably, interpretive conclusions must be viewed as provisional. Rather than demonstrating definitive patterns of effectiveness, the reviewed literature primarily illustrates the types of outcomes examined, the directions of observed change, and the substantial gaps that remain in intervention evidence. Accordingly, this discussion focuses on patterns directly observable in the results, while avoiding causal or structural inferences that are not empirically supported by the included studies.

Across the reviewed interventions, GBV-related outcomes showed modest and uneven responsiveness to community-based, psychosocial, educational, and economic strategies. Several studies reported improvements in intermediate outcomes such as attitudes, psychosocial wellbeing, mobility, or economic indicators, whereas consistent reductions in IPV or sustained changes in women’s autonomy were generally not demonstrated. These patterns suggest cautious promise for selected approaches, while underscoring the limited scope of available evidence regarding long-term, population-level, or equity-oriented impacts.

### Intervention typologies and outcome patterns

Primary prevention interventions, particularly school- and community-based peace education and family-strengthening programs, showed the clearest evidence of change in normative and relational outcomes. One study reported reductions in peer violence and physical punishment, alongside improvements in gender-equitable attitudes among children and adolescents. These findings indicate that interventions targeting younger populations may influence social norms and interpersonal behaviors, although the reviewed evidence does not establish whether such changes translate into sustained reductions in GBV across the life course.

Economic empowerment interventions, most notably the Women for Women International program, were associated with improvements in women’s mobility, savings, food security, and participation in household decision-making. However, these gains were not accompanied by statistically significant reductions in IPV or depressive symptoms. The results, therefore, support a limited conclusion that economic programs may improve selected material and social indicators without, on their own, producing measurable changes in exposure to violence or in mental health outcomes.

Secondary and tertiary interventions addressing psychosocial distress demonstrated mixed mental health effects. The Thinking Healthy Program was associated with significant reductions in postpartum depressive symptoms among participants who completed the intervention, yet attrition exceeded 70%. This high dropout rate, reported in the results, points to challenges in sustaining engagement rather than to demonstrated intervention failure. Similarly, a GBV-focused mental health hotline showed feasibility, acceptability, and cost-effectiveness, with improvements in psychological well-being among users. Importantly, the study also documented that many women relied on shared or male-controlled phones, a factor that constrained privacy and consistent access. This finding indicates that technological delivery platforms may expand reach for some women while simultaneously reproducing existing access inequities.

While the reviewed studies did not directly measure structural violence, insecurity, or governance conditions, several reported findings reflect constraints operating at household, community, and service-delivery levels. For example, community development initiatives increased women’s participation in village activities and income-generating programs but did not produce measurable changes in autonomy, mobility, or intra-household power relations. Similarly, economic and psychosocial interventions demonstrated improvements in selected outcomes without corresponding reductions in IPV. Together, these patterns suggest that many interventions achieved partial or domain-specific effects rather than comprehensive transformations in women’s safety or status.

Across studies, GBV was commonly framed within familial or interpersonal domains, and few interventions reported engagement with formal legal systems, protection mechanisms, or coordinated referral pathways. The absence of evidence on sustained institutional integration limits conclusions about broader system-level change. Thus, rather than asserting that intervention effectiveness is fundamentally shaped by structural violence, the findings more conservatively indicate that existing interventions operate within environments where many structural determinants are not directly addressed by program design or evaluation.

### Implications for interpretation

The evidence supports three bounded conclusions. First, some community-based and psychosocial interventions can influence attitudes, relational behaviors, and mental health symptoms. Second, economic and participatory programs may improve women’s material conditions and social participation without necessarily reducing GBV. Third, the limited number of studies, high attrition in some interventions, and narrow range of measured outcomes restrict inferences about sustainability, scalability, and equity impact.

Future research would benefit from designs that explicitly examine how political context, security conditions, service infrastructure, and legal frameworks interact with program implementation and outcomes. At present, however, the reviewed evidence allows only a cautious interpretation centered on proximal effects rather than definitive claims about structural transformation or long-term reductions in violence.

### Implications for gender and health equity

From an equity perspective, the reviewed interventions reveal uneven benefits across gender, age, and risk groups. Children, adolescents, and women with access to supportive community or health platforms appeared to benefit most, while the most marginalized women, those experiencing severe violence, isolation, or mobility restrictions, were least likely to be reached or retained. This pattern reflects what has been described as equity dilution, wherein interventions disproportionately benefit those already closer to services and social capital.

Notably, few interventions explicitly engaged men and boys as agents of change beyond school-based settings, despite evidence that male attitudes and behaviors are central drivers of GBV. Moreover, the lack of rigorous monitoring and evaluation frameworks limited the ability to assess long-term equity impacts, intersectional vulnerabilities, or unintended harms.

However, given the current political environment in Afghanistan, where restrictions on women’s mobility, employment, and civil society engagement are severe, large-scale structural reform or openly rights-based programming may not be feasible or safe. In this context, equity-oriented action may need to focus on incremental, low-visibility strategies that prioritize harm reduction rather than systemic transformation.

The reviewed evidence suggests that platforms already embedded within routine services, such as maternal health care or community-based education programs, may represent the most realistic entry points for reaching women without increasing exposure to risk. Ensuring confidentiality, minimizing documentation burdens, and protecting frontline female staff are practical equity measures that align with the constraints described in the results.

### Implications for practice, policy, and research

The findings suggest that GBV interventions in Afghanistan showed measurable effects primarily when they were community-embedded or delivered through existing health or education platforms. Primary prevention efforts that integrate education, parenting support, and norm change demonstrated shifts in attitudes and peer violence. Psychosocial interventions and hotlines showed improvements in mental health outcomes, although access was mediated by attrition and technology constraints, including reliance on shared or male-controlled phones.

In the current Afghan context, practical action is likely to be limited to preserving services and mitigating harm rather than expanding them. Maintaining discreet psychosocial support within primary health care, sustaining confidential hotline services where feasible, and embedding GBV-sensitive approaches within broader humanitarian programming may represent the most actionable strategies. These approaches build on interventions that have already demonstrated feasibility in the reviewed studies.

Given the contraction of international donor funding for gender-based violence work, external actors may need to prioritize flexible funding mechanisms that support local health providers and community networks rather than large, highly visible standalone GBV programs. Small-scale, adaptive programming that can operate within shifting political conditions may be more sustainable than externally driven structural initiatives.

At the policy level, the evidence underscores that GBV prevention cannot be separated from broader governance and institutional conditions.

However, in settings where direct policy reform is unlikely, international engagement may need to focus on safeguarding essential health services for women, protecting female health workers, and supporting remote technical assistance rather than advocating for immediate legislative change.

This review also offers lessons for other conflict-affected and politically restricted settings. First, interventions that focus exclusively on economic empowerment without concurrent relational or normative components may not reduce violence, even when economic indicators improve. Second, delivery mechanisms must account for gendered access to communication technologies and mobility. Third, high attrition rates in psychosocial interventions underscore the need to design programs that accommodate insecurity, caregiving burdens, and limited mobility. These lessons are transferable to other fragile contexts where service systems are weak and women’s public participation is limited.

With respect to research, conducting large-scale primary research on violence against women in Afghanistan may currently raise ethical and safety concerns. As a result, secondary data analysis, remote methodologies, partnerships with trusted local providers, and the integration of GBV indicators into existing humanitarian monitoring systems may be more ethically appropriate in the near term.

Future studies should prioritize designs that capture mechanisms and contextual adaptation when conditions allow. In highly restrictive environments, strengthening monitoring within existing service platforms may be more feasible than initiating new experimental trials.

### Limitations

This scoping review has several important limitations that should be considered when interpreting the findings. First, the evidence base on GBV prevention and response interventions in Afghanistan is extremely limited. Only seven intervention-focused studies met the inclusion criteria over 15 years, reflecting both the scarcity of evaluated programs and the profound challenges of conducting intervention research in conflict-affected and politically restrictive settings. Accordingly, this review should be understood as mapping the contours of available evidence rather than estimating intervention effectiveness. As a result, the findings cannot be interpreted as representative of all GBV-related programming implemented in Afghanistan, particularly informal, community-led, or unpublished initiatives.

Second, the included studies exhibited substantial heterogeneity in intervention type, study design, target populations, outcome measures, and theoretical frameworks. This heterogeneity precluded direct comparisons across interventions and limited the ability to draw conclusions about relative effectiveness. Moreover, many studies relied on short follow-up periods, self-reported outcomes, or non-randomized designs, constraining inferences about long-term impact, sustainability, and causal pathways. Observed changes should therefore be interpreted as context-specific and preliminary.

Third, consistent with the methodological purpose of scoping reviews, this study did not include a formal quality appraisal or risk-of-bias assessment of the included studies. While this approach is appropriate for mapping the breadth and nature of existing evidence, it limits the ability to assess the strength or robustness of reported findings. Some interventions reported promising outcomes; however, these results should be interpreted cautiously, given issues such as high attrition, small sample sizes, and contextual disruptions related to insecurity and mobility restrictions.

Fourth, the review is constrained by publication and language bias. Although multiple databases were searched, relevant program evaluations may exist in grey literature, internal NGO reports, or non-English sources that were not captured. Locally led initiatives implemented after 2021 may be underrepresented due to restrictions on documentation, dissemination, and international collaboration.

Finally, the rapidly changing political context in Afghanistan, especially following the Taliban’s return to power in 2021, limits the temporal applicability of some findings. Several interventions were implemented under markedly different governance conditions, and their feasibility, scalability, or ethical viability may no longer be transferable to the current context. Consequently, the review reflects both historical and contemporary evidence, which must be interpreted in light of ongoing institutional collapse, legal regression, and gendered exclusion.

Despite these limitations, this scoping review provides a structured synthesis of a highly fragmented literature and identifies critical gaps in intervention coverage, evaluation, and equity orientation. It offers a cautious foundation for future research and programming rather than definitive guidance on best practices.

## Conclusion

This scoping review demonstrates that, despite a minimal and uneven evidence base, GBV prevention and response interventions in Afghanistan have been associated with select improvements in psychosocial wellbeing, gender attitudes, family functioning, and women’s economic participation, while consistent reductions in violence exposure have not been robustly demonstrated. These findings indicate partial and domain-specific effects rather than comprehensive or sustained change.

The review further indicates that programmatic efforts alone are insufficient to address the conditions in which gender-based violence occurs. Within the limits of the available evidence, interventions appear most viable when they are community-embedded, culturally responsive, and delivered through existing health or education platforms. However, their potential impact remains constrained by broader political, institutional, and social conditions that were largely outside the scope of the evaluated interventions.

Afghanistan represents an extreme yet instructive case for the global GBV prevention field. The evidence synthesized here suggests that in contexts of profound restriction, progress toward gender and health equity is likely to be incremental and fragile, emphasizing harm reduction, service preservation, and protection of women’s access to care rather than rapid transformation.

Advancing this agenda will depend on sustained international engagement, locally led and adaptive approaches, and an evidence base that prioritizes ethical feasibility, equity-sensitive monitoring, and the lived realities of Afghan women and girls.

## Data Availability

All data generated and analyzed during this review are included in the published review article.

## Abbreviations

GBV: Gender-based violence
HIVRR: HIV risk reduction
IGA: Income-generating activities
IPV: Intimate partner violence
MH: Mental health
MF: Microfinance
MHPSS: Mental health and psychosocial support
NSP: National Solidarity Program
PP: Postpartum
PPD: Postnatal depression
RCT: Randomized-control trial
